# Supplementary material for: Knowledge and Perception of and Attitude toward a Premarital Screening Program in Qatar: A Cross-Sectional Study
Source: Int J Environ Res Public Health. 2022 Apr 6;19(7):4418. doi: 10.3390/ijerph19074418 (PMC8998822; doi:10.3390/ijerph19074418)
Supplement: Supplementary file 1 [file ijerph-19-04418-s001.zip › Supplementary S2 Questionnaire.pdf]

Supplementary 2: Questionnaire (S1 Questionnaire)

Knowledge and Attitude of Premarital Screening (PMS)

<u>The questionnaire:</u>

1. What is your age?
    - 18-21
    - 22-25
    - 26-29
    - 30 years or above
2. What is your gender?
    - Male
    - Female
3. What is your nationality? Please specify
    - Qatari
    - Non-Qatari (please specify) ---------------------
4. What is your religion?
    - Islam
    - Christianity
    - Hinduism
    - Buddhism
    - Others, please specify----------------------
5. In which college are you studying at Qatar University?
    - College of Business and Economics
    - College of Sharia (Islamic studies)
    - College of Law
    - College of Engineering
    - College of Arts and Sciences
    - College of Health Sciences
    - College of Medicine
    - College of Education
    - College of Pharmacy
6. What is your current year of study in Qatar University?
    - 1st year
    - 2nd year
    - 3rd year
    - 4th year
    - 5th or more
7. Please indicate your current marital status?
    - Engaged
    - Separated
    - Divorced
    - Widowed
    - Single (never married)
8. What is the type of relation between your parents?
    - First cousins (the couple's parents are brother and sisters)
    - Second cousins (the couple's parents are cousins)
    - More distant relation
    - Not related at all
9. Have you or any of your close family member been diagnosed with a genetic disease?
    - Yes
    - No (go to question 14)

- I do not know

10. Have you ever undergone premarital screening?
    - Yes
    - No
11. What is your household income level excluding the drivers' and maids' income?
    - Less than 10,999 QAR
    - 11,000- 19,999 QAR
    - 20,000 QAR or more
12. Have you heard about "Premarital screening" or "genetic testing"?
    - Yes
    - No
13. If yes, what is the main source of your information?
    - TV
    - Friends
    - Academic course
    - Newspapers
    - Social Media
    - Health care facility
    - Other, please specify --------------
14. Diseases targeted by Premarital Screening include (check all that apply):
    - Genetic diseases
    - Sexually transmitted diseases
    - I do not know
15. Blood diseases targeted by Premarital Screening include (Check all that apply):
    - Sickle cell disease
    - Thalassemia
    - G6PD deficiency
    - Heart disease
    - Diabetes
    - I do not know

Please tell me if you agree or disagree with the following:

16. Consanguinity (marriage between cousins) can increase the chance of having a child with a genetic disease.
    - Strongly Agree
    - Somewhat agree
    - Disagree
    - Strongly Disagree
    - Neutral
17. Premarital screening can reduce the occurrence of genetic diseases.
    - Strongly Agree
    - Somewhat agree
    - Disagree
    - Strongly Disagree
    - Neutral
18. Premarital screening can reduce the occurrence of sexually transmitted diseases.
    - Strongly Agree
    - Somewhat agree
    - Disagree
    - Strongly Disagree
    - Neutral
19. Genetic tests are always 100% accurate.

- Yes
- No
- I do not know

20. A genetic test must be repeated every year because the results may change.
    - Yes
    - No
    - I do not know
21. In case of a family having a genetic disease, this will certainly imply that every member of the family would have the disease.
    - Yes
    - No
    - I do not know
22. Genetic diseases can have a psychological burden on families.
    - Strongly Agree
    - Somewhat agree
    - Disagree
    - Strongly Disagree
    - Neutral
23. Genetic diseases can have an economic burden on families.
    - Strongly Agree
    - Somewhat agree
    - Disagree
    - Strongly Disagree
    - Neutral
24. Are you aware of any Islamic fatwa (edict) approving abortion if a fetus affected by genetic abnormalities?
    - Yes
    - No
    - Not applicable
25. What do you think is the appropriate time for doing genetic testing?
    - In high school
    - Pre-joining a college
    - Before marriage (during engagement)
26. Genetic testing could lead to denial of a marriage for a couple.
    - Strongly agree
    - Somewhat agree
    - Disagree
    - Strongly disagree
    - Neutral
27. Genetic testing will do more harm than good for society.
    - Strongly agree
    - Somewhat agree
    - Disagree
    - Strongly disagree
    - Neutral
28. What would be your response if you were told (after the premarital screening) that there would be a chance of having a child affected with a genetic diseases?
    - Continue engagement and get marry
    - Cancel/discontinue engagement
    - Continue engagement and get marriage for emotional reasons
    - Continue engagement and get marriage for family pressure

29. Law and regulation should ban the marriage if there is a chance of having a child affected with a genetic disease.
    - Strongly agree
    - Somewhat agree
    - Disagree
    - Strongly disagree
    - Neutral
30. I prefer consanguineous marriages (marriage between cousins or relatives).
    - Strongly agree
    - Somewhat agree
    - Disagree
    - Strongly disagree
    - Neutral
31. Premarital screening does not interfere with a belief in destiny.
    - Strongly agree
    - Somewhat agree
    - Disagree
    - Strongly disagree
    - Neutral
32. Which sector of society should be educated about genetic diseases? (check all that apply)
    - Families with genetic diseases genes detected
    - Parents
    - Young people preparing for marriage
    - Teenagers/high school students
33. Have you ever heard about screening for Sexually Transmitted Diseases (STDs) in Qatar?
    - Yes
    - No
    - I do not know
34. What is the source(s) of transmission for AIDS? (Check all that apply)
    - Sexual intercourse
    - Genetically inherited
    - Blood contact
    - I do not know
    - Others, please specify ----------------
35. HIV can be prevented by the following method(s): (Check all that apply)
    - Vaccination
    - Use of condoms
    - Use of pills/drugs
    - Abstinence
    - I do not know
36. Screening for Sexually Transmitted Diseases is necessary before marriage.
    - Strongly agree
    - Somewhat agree
    - Disagree
    - Strongly disagree
    - Neutral
37. If somebody discovered that during premarital that he/she has AIDS, this person should:
    - Hide it from partner
    - Break the relation
    - Inform partner
    - Consult a physician
