# Supplementary material for: Knowledge and Perception of and Attitude toward a Premarital Screening Program in Qatar: A Cross-Sectional Study
Source: Int J Environ Res Public Health. 2022 Apr 6;19(7):4418. doi: 10.3390/ijerph19074418 (PMC8998822; doi:10.3390/ijerph19074418)
Supplement: Supplementary file 1 [file ijerph-19-04418-s001.zip › Supplementary S1 Focus group questions.pdf]

Supplementary S1: Focus group questions

Opening question:

Are genetic diseases important health problem in Qatar? Why? What could be associated factors?

1) Perceive benefits:

1- What is your perception about premarital screening? Why it is done? What does it screen for? Where it is done?

2- Do you think that premarital screening is a good practice? why?

2) Perceived susceptibility:

1- Do you think that results of premarital screening will affect your marriage decision? How? To what extent?

2- Do you think your future child will have a higher chance for having some genetic diseases if you and your partner do not do the premarital screening test?

3) Perceived barriers:

1- What could be a concern for you doing premarital screening?

2- Do you think doing premarital screening will prevent you from getting married if the results showed that your future child has a chance to have genetic disease?

3- Do you think doing premarital screening interfere with religion? How?

4) Perceive severity:

1- If you got married without doing PMS and found that some of your children could have a chance for genetic diseases, what would you do? Would you think of getting divorce?

2- Would you agree on abortion if you find out that your expected child would have a genetic disease?

3- Do you believe that you and your future spouse could handle financial or emotional problems due to having a child with a genetic disease?

4- What do you think about marriage between partners if PMS revealed that one of them is infected with HIV? Why?

5) Cues to action:

1. What would you advise a friend whose premarital screening test shows a chance of having a child with genetic disease? What would be your action if you have this similar situation?
2. Do you think that there should be a law that forbid marriage if the PMS have shown that there is a probability to have a kid affected by a genetic disease?
